# Occupational Therapy Using Coping Lists After Total Knee Arthroplasty: A Case Series

**DOI:** 10.7759/cureus.27374

**Published:** 2022-07-27

**Authors:** Ryusei Hara, Yuki Hiraga, Yoshiyuki Hirakawa

**Affiliations:** 1 Department of Rehabilitation, Fukuoka Rehabilitation Hospital, Fukuoka, JPN; 2 Department of Health Care Administration and Management, Graduate School of Kyushu University, Fukuoka, JPN; 3 Department of Health Sciences, International University of Health and Welfare Graduate School, Fukuoka, JPN; 4 Department of Occupational Therapy, Fukuoka International University of Health and Welfare, Fukuoka, JPN

**Keywords:** total knee arthroplasty, psychological factor, pain, occupational therapy, coping list

## Abstract

Total knee arthroplasty (TKA) can improve the postoperative quality of life in patients with severe knee osteoarthritis. Although occupational therapy (OT) using a coping list may be useful for post-TKA patients, its use has not been documented. This study aimed to explore the effectiveness of OT using coping skills. Five post-TKA patients underwent OT using coping skills. The Canadian Occupational Performance Measure (COPM), numerical rating scale (NRS), Hospital Anxiety and Depression Scale (HADS), EQ-5D (EuroQol-5-dimension)-5-level (5L), EQ-5D Visual Analogue Scale (VAS), modified fall efficacy scale (MFES), Pain Disability Assessment Scale (PDAS), and coping skills were measured at the start and end of the study. Significant improvements were observed in COPM, NRS, HADS, EQ-5D-5L, and PDAS scores (p <0.05). No significant improvements were found in the EQ-5D VAS and MFES scores. All evaluations showed a large effect size (r ≤ 0.5). The total number of coping skills also increased. This report suggests that OT with coping strategies is effective for pain, psychological factors, quality of life, and activities of daily living. Incorporating coping skills in OT may be useful in postoperative TKA pain management. However, larger studies are needed to validate this.

## Introduction

Chronic pain is defined by the International Association for the Study of Pain as “an unpleasant sensory and emotional experience associated with, or resembling that associated with, actual or potential tissue damage” [1]. The economic loss due to chronic pain in Japan is estimated to be 1,935 billion yen, and those with chronic pain reportedly experience high levels of psychological distress and a significantly low quality of life (QOL) [2]. Catastrophic thinking, anxiety, and depression have been reported as psychological factors that lead to chronic pain [3]. Therefore, the development of occupational therapy (OT) practices based on psychological factors in the area of chronic pain is urgently warranted.

Knee osteoarthritis (OA) is a disease that typically presents as joint pain, and total knee arthroplasty (TKA) has been shown to improve postoperative QOL [4]. However, it has been reported that approximately 20% of post-TKA patients develop chronic pain, which affects activities of daily living (ADL) and participation in social activities [5,6]. In the early postoperative period, pain mediates anxiety, and self-efficacy affects long-term postoperative life disorders. This suggests that interventions for pain, anxiety, and self-efficacy, within this time frame, are important [7]. Pain-catastrophizing, depression, and lower psychosocial QOL scores among patients who have undergone TKA are associated with the risk of severe pain [5]. Addressing catastrophic effects of pain on psychological aspects, ADL and QOL are therefore crucial.

In recent years, cognitive behavioral therapy (CBT) has been shown to be an effective intervention for pain and psychological disturbances, in patients with chronic pain [8]. Studies on postoperative patients with knee OA, including those who have undergone TKA, have reported improvement of pain and its psychological impact, by practicing OT using coping skills, which is one of the typical techniques of CBT [9]. In addition, there is a practical report that OT using coping skills was found to improve the Canadian Occupational Performance Measure (COPM), which is a measure of goal achievement [9]. Coping skills are described as “various efforts that individuals make to improve the unpleasant situation of pain” [9]. With respect to all of the above, it is evident that achieving appropriate pain management through effective coping skills, from the early postoperative period onward, can break the vicious circle of chronic pain, and lead to an improvement in QOL. However, in Japan, OT interventions that incorporate coping skills for post-TKA patients are not standardized. In addition, it is necessary to verify OT practices using coping skills in a case series format, first. Therefore, this study aimed to explore the effectiveness of incorporating coping skills in OT practices.

## Case presentation

Materials and methods

Study Design

This study was a case series that assessed each evaluation index (COPM, pain, psychological factors; catastrophizing, anxiety, depression, self-efficacy, life disability, QOL), at the start and end of OT.

Ethical Considerations

All patients provided written informed consent to participate in the study. The study design was approved by the ethics review board of Fukuoka Rehabilitation Hospital (FRH-2020-R015).

Participants

Patients who underwent TKA from July 2020 to July 2021 at the institution were included in the study (Table 1).

**Table 1:** Patients’ characteristics

Case	Surgical side	Sex	Age	Hospital stay	Intervention period	Discharge destination
A	Right	female	60	37	28	Home
B	Left	female	70	37	22	Home
C	Right	female	80	52	36	Home
D	Right	female	70	44	28	Home
E	Left	female	60	47	33	Home

Exclusion criteria included a diagnosis of dementia or mental illness (e.g., depression) that would interfere with the completion of the questionnaire, as well as refusal to participate in post-surgical rehabilitation. Additional exclusion criteria were postoperative complications (e.g., nerve injury or deep vein thrombosis), other significant medical diseases interfering with postoperative rehabilitation, previous TKA (e.g., TKA of the opposite limb or revision surgery), and TKA performed for causes other than degenerative diseases (e.g., rheumatoid arthritis or bone necrosis). The screening was performed by an orthopedic surgeon prior to surgery. The TKA surgeries were performed by four surgeons.

Postoperative Rehabilitation

Surgeries were performed under general anesthesia in all patients. All patients received nonsteroidal anti-inflammatory drugs (NSAIDs) (dose, 60 mg, three tablets per day) for two weeks, postoperatively. All patients followed the same physical therapy protocol after surgery. All patients began physical therapy on postoperative day 1, including knee range-of-motion exercises (flexion-extension) and stretching. Approximately 3 weeks post-operation, walking using a walker was started. Approximately 5 weeks postoperatively, walking with a cane or without assistance was possible, and the patient was discharged from the hospital. All physical therapy interventions lasted 40 min/day.

Intervention

Overview of Occupational Therapy Practice

The American Occupational Therapy Association explained that, in OT for pain, one must “implement a self-management approach focusing on participating in daily life” (e.g., set goals for management), “set individual occupational therapy goals,” and start “activation of behavior,” and perform “home exercise” (e.g., management of pain at home) [10].

It has been shown that interventions for OT education, OT goal setting using COPM, and behavioral activation using an activity diary are also effective in Japan [11]. However, there are no reports on pain management at home, which is referred to as home exercise. Therefore, to promote participation in daily activities after discharge, we considered the importance of early pain management in the early TKA postoperative period and devised the following outline.

The treatment time was two sessions twenty minutes each (20 min × 2 units of 40 min). Movement practice was performed step by step, in consultation with the physical therapist, according to the movement form.

Interview Using COPM

The first interview was conducted using COPM, and emphasis was placed on creating an environment in which the interviewer could easily form sympathetic and supportive relationships with the patient while listening to them narrate details of their current pain and anxiety. Further, we listened to the background of the patient’s life before surgery (e.g., daily/weekly schedule, etc.) and determined the necessary activities they needed to fulfill after discharge. We proposed a coping list for the necessary activities needed upon discharge, for the achievement of goals (ADLs, instrumental ADLs, and return to work and applied movements), and for pain management. The coping list included those who had an agreement on introduction and research and those who had an intention to acquire coping skills. At the time of the first interview, measurements of numerical rating scale (NRS), Hospital Anxiety and Depression Scale (HADS), modified fall efficacy scale (MFES), Pain Disability Assessment Scale (PDAS), EuroQol-5-dimension-5-level (EQ-5D-5L), and EQ-5D visual analogue scale (VAS) were performed simultaneously.

Acquisition of Coping Skills

In the early stage of the OT practice, an interview using a coping list was conducted, in combination with OT centered on motion practice (mainly ADL practice, such as bathing motion and step practice), to promote an understanding of its use. We conducted a review to note additional coping skills for patients who could provide specific coping mechanisms. In addition, for patients who lacked specific coping skills, coping skills based on the patient’s hobbies and tastes, which stemmed from coping skills that had already been brought up (for example, coping skills such as “stretching the knees” “stretching the shoulders”), presenting a collection of coping tips, listening to the conditions of the previous day, and acquiring coping skills from pain-free movements and the activities performed during that time, were urged upon. After being given specific coping skills, the patients were recommended to increase the number of coping skills, themselves, for better self-management.

OT Self-management

After progressing to self-management, we reviewed the content that was tackled at the time of the intervention. During the intervention, in addition to ADL practice, instrumental ADLs (activities necessary for life after discharge; for example, cooking, cleaning, shopping, etc.), and outdoor walking practice were carried out, step by step, toward discharge. Finally, an interview using COPM was conducted at the time of the final intervention, an intervention in the form of coping skills was reviewed, and the OT was completed.

At this time, NRS, HADS, MFSE, PDAS, EQ-5D-5L, and EQ-5D VAS were also measured.

Outcomes

Canadian Occupational Performance Measure

OT sessions were performed using the COPM [12]. Using COPM, we recorded the top five patient goals and prioritized them according to their importance. Subsequently, for each goal, the degrees of performance and satisfaction were evaluated using the 10-case method, and the average value of each item was calculated. The patients also practiced movements involved in their ADLs and instrumental ADLs (including cooking, cleaning, shopping, etc.), which were important for achieving goals and enabling hospital discharge.

Coping Skills

**Figure 1:**
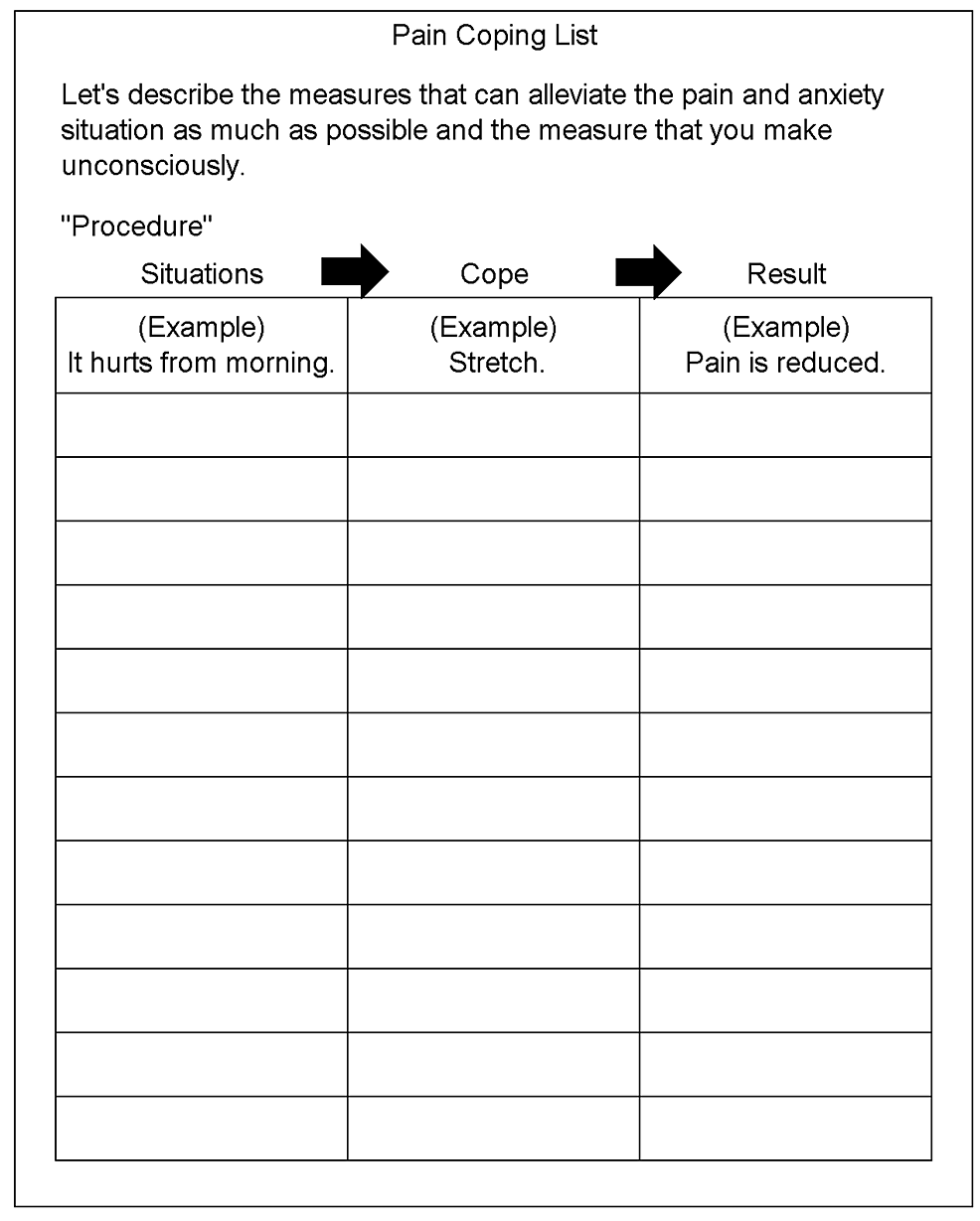
Coping list The strategy is to describe how to deal with pain and anxiety in daily life and what kind of results were obtained.

Regarding coping skills, a coping list (Figure 1) was used [9]. A coping list is a tool that describes the kind of coping that should be used for pain and anxiety situations and the kind of results that were obtained. The total number of effective coping skills for the adopted coping was tallied.

Pain

An NRS was used to evaluate pain [13]. The 11-point scale ranged from a grade of 0, which corresponded to “no pain”, to 10, which corresponded to “unbearable pain”.

Anxiety and Depression

The HADS was used to evaluate anxiety and depression [14]. The HADS is a self-administered questionnaire and consists of 14 questions and two scales-one for anxiety and the other for depression. A score of 0 to 7 points is considered as “no anxiety/depression,” 8 to 10 points are considered “suspicious for anxiety/depression,” and 11 points or more is considered “confirmed anxiety/depression”.

Self-efficacy for Daily Life

The MFES was used to evaluate self-efficacy in daily life [15,16]. The MFES is a self-administered questionnaire that consists of 14 questions, including those on ADLs and instrumental ADLs. It was developed as a fall-evaluation tool for the elderly and is correlated with self-efficacy in daily life [15,16]. Each item can be given one of 11 ratings, ranging from 0 (“not confident”) to 10 (“completely confident”). Higher scores reflect a higher self-efficacy for daily life.

Disability for Pain

The PDAS was used to evaluate life disorders associated with pain [17]. The PDAS is a self-administered questionnaire used to measure life disorders associated with chronic pain. It is a four-case method consisting of 20 items, with each item graded from 0 to 3. Higher scores indicate stronger disabilities. The cut-off value was set at 10 points.

QOL

QOL was measured by the EQ-5D-5L questionnaire, which contains five questions with five responses for each question, and the total score is converted into the final EQ-5D value, ranging from 0.000 to 1.000; higher scores indicate a better QOL [18]. The EQ-5D questionnaire also includes a VAS, by which respondents can report their perceived health status with a grade ranging from 0 (the worst possible health status) to 100 (the best possible health status) [18].

Data analysis

Statistical analyses were performed using JMP software version 14.2.0 (SAS Institute Co.), Ltd, and descriptive statistics were used to describe the demographic data. Descriptive analyses were performed using mean, standard deviation, and frequencies. Pre-OT and post-OT data were compared in terms of outcomes (COPM, NRS, HADS, EQ-5D, EQ-5D VAS, MFES, PDAS) using the Mann-Whitney U test with JUMP 14.2.0 (SAS Institute Co., Ltd). The effect size (r) to describe the magnitude of the treatment effect was as follows: small, 0.10 to < 0.30; medium, 0.30 to < 0.50; and large, ≥ 0.50 [19].

Results/findings

The values of each evaluation index at the start and end of the OT are shown in Table 2. Significant improvements were observed in the COPM, NRS, HADS, PDAS, and EQ-5D-5L (P < 0.05) (Table 3). No significant improvement was found in the EQ-5D VAS and MFES scores. The effect size (r) of each evaluation was r ≥ 0.5, indicating a large effect size.

**Table 2:** Pre-and post-OT outcomes, by cases Values are expressed as mean ± standard deviation. OT: occupational therapy; COPM: Canadian Occupational Performance Measure; NRS: numeric rating scale; HADS: Hospital Anxiety and Depression Scale; EQ-5D-5L: EuroQol 5 dimensions 5-level; VAS: Visual analog scale; MFES: Modified Falls Efficacy Scale Modified Falls Efficacy Scale; PDAS: Pain Disability Assessment Scale

	Case A		Case B		Case C		Case D		Case E	
	Pre-OT	Post-OT	Pre-OT	Post-OT	Pre-OT	Post-OT	Pre-OT	Post-OT	Pre-OT	Post-OT
COPM-performance	1	8	5	7	3	10	2	10	1	8
COPM-satisfaction	1	8	5	7	3	10	1	10	1	8
NRS	5	0	4	1	6	1	7	1	10	4
HADS depression	5	2	5	3	9	1	12	0	11	2
HADS anxiety	5	4	4	3	4	1	9	4	9	3
EQ-5D	0.6	1	0.8	0.8	0.7	0.8	0.2	0.8	0.3	0.8
EQ-5D VAS	80	90	75	90	50	70	70	70	30	80
MFES	76	138	102	2	132	139	53	96	105	110
PDAS	28	6	34	28	16	4	13	5	40	13
Coping skill (number)	28		23		21		11		13	
Job and role	Farmer		Housewife		Housewife		Housewife		Housewife	
Current anxiety	Return to work		Pain		Pain		Life after discharge		Pain and gait	

**Table 3:** Pre-and post-OT outcomes, by case group Values are expressed as mean ± standard deviation. OT: occupational therapy; COPM: Canadian Occupational Performance Measure; NRS: numeric rating scale; HADS: Hospital Anxiety and Depression Scale; EQ-5D-5L: EuroQol 5 dimensions 5-level; VAS: Visual analog scale; MFES: Modified Falls Efficacy Scale Modified Falls Efficacy Scale; PDAS: Pain Disability Assessment Scale. *Significant difference between pre-OT and post-OT (p < 0.05). **Significant difference between pre-OT and post-OT (p < 0.01)

	Pre-OT	Post-OT	p-value	Effect size (r)
COPM-performance	2.4±1.5	8.6±1.2	0.01**	1.2
COPM-satisfaction	2.2±1.6	8.6±1.3	0.01**	1.2
NRS	6.4±2.1	1.4±1.4	0.01**	1.1
HADS depression	8.4±2.9	1.6±1.0	0.01**	1.2
HADS anxiety	6.2±2.3	3±1.1	0.03*	1.0
EQ-5D	0.5±0.2	0.8±0.1	0.02*	1.1
EQ-5D VAS	61±18.5	80±8.9	0.14	0.7
MFES	93.6±27.0	118±17.4	0.12	0.7
PDAS	26.2±10.3	11.2±9.0	0.05*	0.9

In addition, the total number of coping skills that were effective for each patient increased (Table 4). All participants had positive feedback at the time of discharge.

**Table 4:** Acquired coping skills, by case

Case A	Case B	Case C	Case D	Case E
Take a deep breath	Taking medicine	Icing	Icing	Icing
calm down	Rehabilitation	Be positive	Taking medicine	Talk about pain
Prepare	Stretch	Have a goal	Hot pack l	Contact with family
Disperse feelings	Rest	Rehabilitation	TV set	Medication management
Have room	Icing	Listen to music	Eat sweets	Be positive
Think good	Looking out	Relaxation	Distract attention from pain	Get sleep
Not think of anything	Listen to music	Walking	Rehabilitation	Blame someone
Distract attention	Hot pack	Medication management	Stretch	Talk to people
Relax	Talk to the patient	Talk to a nurse	Take a break	Eat what you like
Icing	Phone with family	Inhale the outside air	Don’t think about pain	Watch TV
Positioning	Think about what you want to eat	Strength training	Encourage yourself	Become defiant
Stretch	Think about life after discharge	To sew		Positioning
Strength training	Schedule	Take a bath		Ignore pain
Walking	Talk to the therapist	Stretch		
Sleep	Write a diary	Radio gymnastics		
Take medicine	Look at the foliage plants	Do yoga		
Gymnastics	watch TV	Phone with family		
Contact with family	Drink coffee	Talk		
Talk to other patients	Fabric shaver	Clean		
Write a diary	Play a jigsaw puzzle	Reading		
Tell a dream	Walking	Make accessories		
Reading				
Rest				
Talk to the therapist				
Looking out				
Relax your body				
Have a goal				
Exercise (stairs)				

Progress of OT among patients

The progress of patients in terms of coping skills is listed below.

Case A: Anxiety was noted for pain and climbing stairs. After acquiring coping skills, he was able to manage pain and climb stairs and was discharged from the hospital after acquiring a total of 28 effective coping skills and saying, “I have no particular anxiety.”

Case B: The patient was anxious about pain. After acquiring coping skills, pain management became possible. The patient said, “I’m glad that I felt like doing this in various situations,” and was discharged from the hospital after acquiring a total of 23 effective coping skills.

Case C: Anxiety about pain was noted. After the introduction, it was difficult to improve effective coping skills; therefore, in the first week of the intervention, we deepened our understanding of their use, mainly through interviews. Effective coping skills were mentioned on the 5th day. After that, the patient acquired a total of 21 effective coping skills, said, “I am looking forward to my future life,” and was discharged from the hospital.

Case D: Pain and anxiety in life after discharge were noted. After acquiring coping skills, the patient reported subsidence in pain, acquired 11 effective coping skills, and was discharged from the hospital.

Case E: The patient became inactive due to anxiety caused by pain and admitted that he wanted to be anesthetized to feel better, even for a day. Through the interviews, we encouraged the acquisition of coping skills through empathic and supportive relationships. After the introduction, he acquired 13 effective coping skills and said “I think I can live at home,” and was discharged from the hospital.

## Discussion

Occupational therapy practice using coping skills

In this case series, improvements in COPM, NRS, HADS, and EQ-5D-5L were observed in five post-TKA patients, by OT practice using a coping list. To the best of our knowledge, this is the first case series combining coping lists and OT practice, after TKA. In a study, Riddle et al. reported that the practice of incorporating coping skills was found to improve pain, living function, and catastrophic pain in post-TKA patients, after two months [20]. Furthermore, a significant improvement in physical function was observed, compared to patients in the control group [20]. Similarly, in this study, significant improvements in pain and living function were observed with OT practice incorporating coping skills.

In another cohort study, Riddle et al. presented a practical protocol based on previous studies, incorporating coping skills after TKA [20]. This study compared programs designed with a focus on general physical rehabilitation and pain coping, and the presented protocol included pain, physical function and activity, QOL, pain management, and psychological factors. From these studies, it may be considered that the practice of incorporating coping skills in OT can lead to improvements in pain, QOL, psychological factors, and so on. Additionally, in this study, pain, psychological factors, disability, and QOL were improved by acquiring coping strategies, using the coping list, and connecting them to self-management.

However, this intervention did not result in a significant improvement in MFES. It has been reported that higher levels of self-efficacy in acquiring coping skills result in a greater effect; thus, MFES plays an important role in coping with pain [8]. Our results can likely be explained by the fact that there were many cases in which the MFES score and self-efficacy were higher at the end than at the start of the intervention. It is probable that these results were obtained because they were practiced in cases where the intention to acquire one’s own coping skills was obtained at the introduction.

Clinical application

According to a survey conducted among elderly people with long-term pain, patients felt that they did not understand the pain, they were not interested in their own pain, and they gave up on the situation of pain [8]. Therefore, it is important to deepen relationships through goal setting and working together to solve problems [9]. It is expected that intervention using coping skills will lead to smooth goal achievement and improve pain management and QOL after discharge. However, since techniques involving self-management, such as coping skills, strongly reflect the will of the individual, it can be said that interventions for post-TKA patients who are not driven remain problematic.

Limitations and prospects of this research

Since this study was a case series (small sample size), the results cannot be generalized. In addition, the long-term effects are unknown because of the limitations of the OT practiced by the first author and the verification of short-term effects alone. Furthermore, because physical therapy and drug therapy are used in combination, the confounding effects of these cannot be completely eliminated.

## Conclusions

This report suggests that OT with coping skills is effective for pain, psychological factors, quality of life, and living function. Incorporating coping skills in OT may be useful in post-TKA/TKR pain management. However, larger studies are needed to validate this. Furthermore, because physical therapy and drug therapy are used in combination, the confounding effects of these cannot be completely eliminated.
